# Biogeography and Conservation in the Arabian Peninsula: A Present Perspective

**DOI:** 10.3390/plants13152091

**Published:** 2024-07-28

**Authors:** Shahina A. Ghazanfar

**Affiliations:** Royal Botanic Gardens Kew, Richmond TW9 3AE, UK; s.ghazanfar@kew.org

**Keywords:** Arabian Peninsula, biogeography, conservation, crop wild relatives, ecoregions, endemism, floristics, genetic resources

## Abstract

The Arabian Peninsula, with its rugged mountains, wadis, alluvial plains, sand dune deserts, and diverse coastlines, spans over 3 million km^2^. The Peninsula is situated at the crossroads of Africa and Asia and is a meeting point for diverse biogeographic realms, including the Palearctic, Afrotropical, and Indomalayan regions. This convergence of biogeographic zones has resulted in a remarkably diverse flora and fauna, which is adapted to the harsh and varied climates found throughout the Peninsula. Each of the countries of the Arabian Peninsula are biologically diverse and unique in their own right, but Yemen, Saudi Arabia, and Oman are the most diverse in terms of their landforms and biological diversity. The mountainous regions support a cooler and more moderate climate compared to the surrounding lowlands, thus forming unique ecosystems that function as refugia for plant and animal species, and have a high endemism of plant species. The desert ecosystems support a variety of lifeforms that are specially adapted to an extreme arid climate. Due to its long history of human habitation and subsistence agriculture, particularly in the mountainous areas, the Arabian Peninsula possesses unique crop varieties adapted to extreme arid climates, making them important genetic resources for the future in the face of climate change. The Arabian Peninsula, though rich and diverse in its biological diversity, has been greatly affected by human activities, especially in the last 50 years, including urbanization, habitat destruction, overgrazing, and climate change, which pose significant threats to the biodiversity of the region. This review presents the biogeography and background of conservation efforts made in the countries in the Arabian Peninsula and gives the progress made in botanical research and conservation practices throughout the Peninsula.

## 1. Introduction

**Figure 1:**
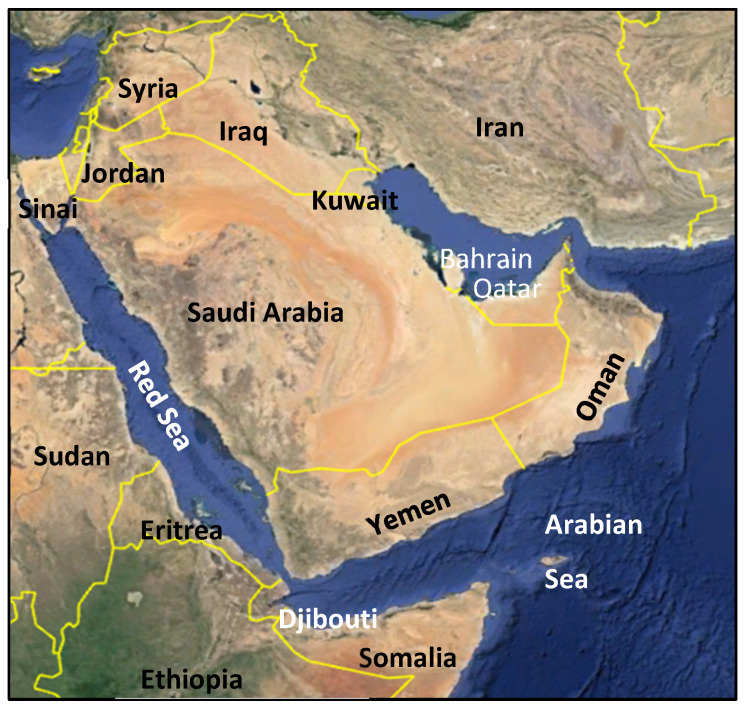
Map of the Arabian Peninsula and surrounding countries.

The Arabian Peninsula lies in the southwestern part of Asia and includes the countries of Bahrain, Kuwait, Oman, Qatar, Saudi Arabia, the United Arab Emirates (UAE), and Yemen. It lies approximately 12 °N (southern Yemen) to 32 °N (northern Saudi Arabia) and about 34 °E (northwestern Saudi Arabia) to 60 °E (eastern Oman) (Figure 1). The area of the Peninsula is characterized by mountains, wadis, plains, and gravel and sand deserts. Its geographical position, between Africa and Asia, is reflected in its biogeography, having elements of both Africa and Asia [1]. Despite its arid conditions, the Arabian Peninsula is home to a variety of plant species; many of these are endemic, and many taxa are relictual from a former wider distribution, now contracted to niches in the mountains where the climate is relatively cooler and more humid than on the plains [2].

The rapid development of the countries of the Arabian Peninsula over the last 50 years neglected the conservation of unique and fragile ecosystems with the result that large areas of the Peninsula are degraded due to overgrazing by livestock and urban development. Efforts are now underway to conserve and protect important plant areas and to restore degraded land.

In this review, along with the background knowledge of the biogeography and conservation of the Arabian Peninsula, contributions from new research are given that add towards our understanding of the biogeography of the Peninsula. The review also provides information on research and efforts that are being taken to conserve the biological diversity and restore degraded landscapes.

### 1.1. Geology and Geomorphology

The region of the Arabian Peninsula is characterized by its diverse geology and geomorphology, which is reflected by its ancient geological history. Ancient shields formed in the Precambrian (4600 million years ago–541 (+/−1) million years ago) represented by ancient igneous and metamorphic rocks include the Arabian–Nubian Shield in the west of the Peninsula and the Tuwaiq Mountains in Saudi Arabia [3,4,5]. Tectonic activity has resulted in the formation of the Red Sea and the Gulf of Aden in the western and southern parts of the Arabian Peninsula and tectonic shifts and volcanic activities have resulted in areas such as Harrat ar Rahat in Saudi Arabia [6,7]. Large parts of the Arabian Peninsula are covered by sedimentary rocks and basins, including the Rub’ al Khali in the southeast and the Persian Gulf region in the northwest [8].

The different landforms of the Arabian Peninsula include its mountains, plateaus, wadis, deserts, and coastlines. The mountains range in elevation from 400 to 3660 m; these include the Asir Mountains in the south of the Peninsula and the Hijaz Mountains in the west (which also includes the Sarawat Mountains of SW Yemen). These mountain ranges run along the Red Sea and include the highest peak, Jabal Shuayb, in Yemen at 3660 m a.s.l. The Hajar Mountains in northern Oman and the Dhofar Mountains in southern Oman range in elevation from 400 to 3009 m. The highest peak, Jabal Shams at 3009 m, lies in the northern Hajar Mountains. The mountains are dissected by gorges and wadis that flow in the plains as alluvial fans. The wadis, with the exception of a few, though dry for most of the year, flow during periods of rainfall. The central region of the Arabian Peninsula is dominated by the Nejd Plateau. It is an elevated, generally flat area with low hills ranging in elevation from 500 to 1000 m a.s.l. [4]. The Nejd Plateau has been inhabited for millennia, historically in its oases, and by pastoral communities. It has many archeological sites, petroglyphs, and pre-Islamic structures, indicating it as a region of trade routes, and it is of historical and cultural significance [9].

The deserts areas, formed during periods of climate change, are major landscapes of the Arabian Peninsula. The Rub’ al Khali occupies large areas of Saudi Arabia, Oman, the UAE, and Yemen. It is a dune desert with gravel areas and sand dunes that reach to elevations of over 250 m. Other deserts, An Nafud in the north central part of Saudi Arabia and Ad Dhana in the south, are characterized by sand dunes and gravel plains. The An Nafud, with its sand dunes, plateaus, and wadis, forms an ecological barrier separating the eastern and central parts of the Arabian Peninsula. 

The Arabian Peninsula has a coastline of approximately 9179 km, spanning several countries; it is characterized by sandy beaches, cliffs, and rocky shores. Mangroves and coral reefs are present in some areas, especially along the coast of Oman and Yemen and along the Red Sea in Saudi Arabia.

Historically and at present, the coasts of the Arabian Peninsula have vibrant trading ports and harbors. Mangroves (*Avicennia marina*) and many halophytic species are found along the coasts of all countries of the Peninsula [10].

### 1.2. Climate

Throughout its geological history, the Arabian Peninsula has experienced climatic fluctuations that have greatly influenced its landscapes and ecosystems. During the Pleistocene (2.6 million to 11,700 years ago), alternated glacial and interglacial periods resulted in arid and humid conditions [11]. These periods are evident from sediment cores from ancient lake deposits, fossilized fauna, and ancient soil layers. After the last glaciation, during the Holocene (11,700 years ago to present), the onset of warmer conditions started over the Arabian Peninsula [12]. During the early Holocene, a more favorable climate than now prevailed with increased precipitation and expansion of vegetation cover. Archeological sites indicate human occupation, with evidence of agricultural practices [13]. The climate, over time, gradually became arid, leading to the formation of the desert landscapes we see today. The vegetation cover decreased, favoring xeric trees and shrubs that are adapted to arid environments. This is evidenced from the presence of many succulent species, species inhabiting specialized niches, and relictual taxa found throughout the Arabian Peninsula.

The present climate of the Arabian Peninsula is characterized by its complex topography and atmospheric circulation and exhibits significant spatial variations [14]. According to the Koppen–Geiger climate classification [15], the climate is classified as a ‘desert climate’ with highs of up to 45 °C in the deserts to lows of 2 °C on the highest summits of Yemen, Saudi Arabia, and Oman. Winter temperatures in the lower hills and plains are usually recorded during November to January with an average air temperature of about 22 °C. Spring and winter seasons typically experience the highest levels of precipitation, respectively. In general, lower temperatures are in the north of the Peninsula and higher temperatures are reported southwards [14]. Throughout the summer, small quantities of precipitation are recorded, while the autumn season receives more precipitation than the summer season. Despite the low mean annual rainfall, isolated storm events characterized by high-intensity rainfall occur from time to time. These are typically recorded during the winter months when weather systems move from west to east, rising into the mountainous areas. According to the analysis of rainfall data recorded at the Tabuk Gauging Station (NW Saudi Arabia), a 6 h duration storm will yield approximately 60 mm of rainfall on average once every 50 years, with the possibility of a rainfall intensity of almost 100 mm/hour during the peak of the storm [16]. The intensity of rainfall events, coupled with sparse vegetation cover, often results in flash flooding.

Rainfall patterns vary greatly from place to place in the Arabian Peninsula. Comparing climatic data from the recent past (1994–2009) and long-term data (>30 years: 1979–2009) at station level over Saudi Arabia shows a significant decrease in rainfall and an increase in mean temperature. However, it is shown that rainfall has increased in the south of the Arabian Peninsula and, in particular, along the Red Sea coast in recent decades compared with that in the 1980s. However, these changes in rainfall are different from results seen inland, where rainfall shows no difference [16].

## 2. Ecoregions and Plant Diversity

There are approximately 4000 plant taxa in the Arabian Peninsula with endemism estimated to be about 20% (c. 800 taxa) [17] (Table 1). The majority of plants are present in the mountains and lower hills of Yemen, Saudi Arabia, and Oman. Country-wise, Yemen, Saudi Arabia, and Oman are the richest in plant taxa and also have the highest endemism (Table 1). Several Oman endemics extend their distribution to the eastern mountains of the United Arab Emirates (UAE).

**Figure 2:**
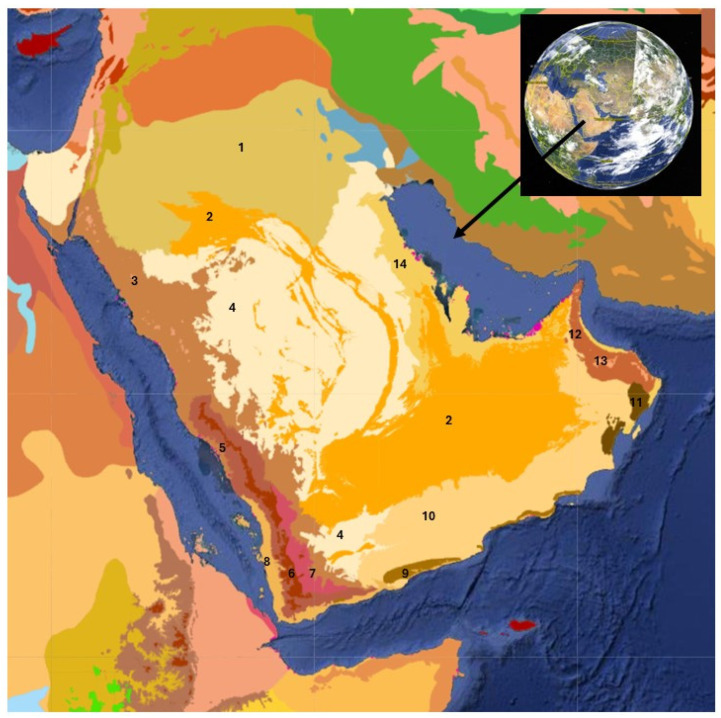
Ecoregion map of the Arabian Peninsula. Adapted from Dinerstein et al. [18]. 1 North Arabian Desert; 2 Arabian Desert; 3 Red Sea–Arabian Desert shrubland; 4 Arabian Sand Desert; 5 Southwest Arabian Escarpment shrublands and woodlands; 6 Southwest Arabian montane woodlands and grassland; 7 Southwest Arabian highland xeric scrub; 8 Southwest Arabian coastal xeric shrublands; 9 South Arabian fog woodlands, shrublands, and dunes; 10 South Arabian plains and plateau desert; 11 East Arabian fog shrublands and sand desert; 12 Al-Hajar xeric woodland and shrubland; 13 Al-Hajar montane woodland and shrubland; 14 Arabian–Persian Gulf coastal plain desert.

**Figure 3:**
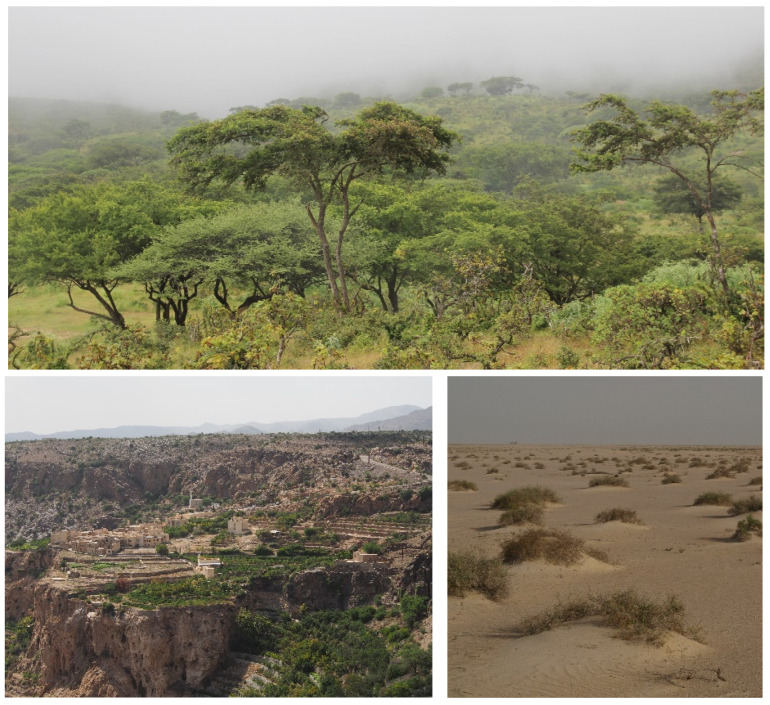

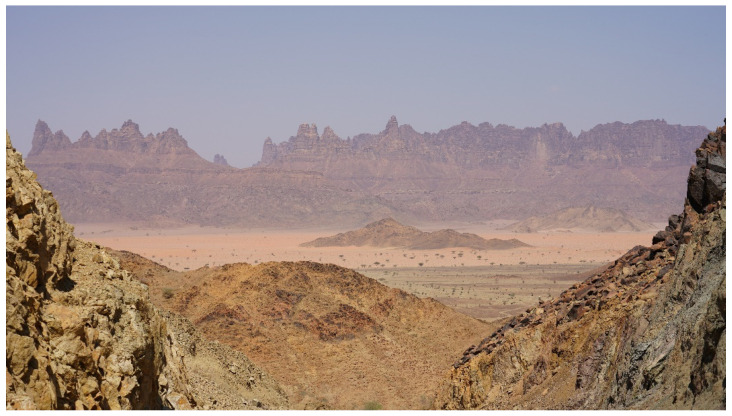
**Top**: Southern mountains of Oman with an open deciduous woodland dominated by *Terminalia dhofarica,* photographed in the monsoon season. **Middle L**. Terraced cultivation of pomegranate, lime, and vegetables in the northern mountains of Oman. **Middle R**. Northwest desert of the United Arab Emirates with *Haloxylon salicornicum* on low sand mounds. **Below**: Sandstone mountains in NW Saudi Arabia with *Vachellia tortilis* at base. Photos: © S.A. Ghazanfar.

The ecoregions of the Arabian Peninsula were outlined in Dinerstein et al. [18] (Figure 2 and Figure 3). In general terms, the vegetation of the mountains of the Arabian Peninsula consists of open montane and deciduous woodlands, deciduous and semi-evergreen shrublands, and grasslands. Xerophytic plant communities on the slopes and rocky summits are common on the mountains. Three species of *Juniperus*, *J. phoenicea*, *J. procera,* and *J. seravschanica*, occur on the montane regions of the Peninsula. *J. phoenicea* is a Mediterranean element found in western Saudi Arabia, while *J. procera* is distributed in the southwest and west of the Arabian Peninsula in Yemen and Saudi Arabia. It is sympatric with *J. phoenicea* along the escarpment mountains in the vicinity of Taif. *J. seravschanica*, an Irano-Turanian species, which occurs in the western Hajar range of the northern mountains of Oman [19,20]. These three species of *Juniperus* are at their outmost posts in the mountains of the Arabian Peninsula and, in this location, are relictual taxa in a state of decline [21,22].

Drought-deciduous *Vachellia* (*Acacia*) and semi-evergreen *Olea europaea* open woodlands are the dominant vegetation over the lower altitudes of most of the western, southwestern, and eastern mountains of the Peninsula [23]. In Yemen and southern Oman, *Vachellia* is associated with *Commiphora* to form a mosaic of open *Vachellia–Commiphora* shrubland. Many other species of shrubs and trees are associated with *Vachellia* and *Commiphora*, in different parts of the Peninsula, including several succulent communities that are found mainly in rocky areas. Orophytic grasslands are present in the montane regions of the Arabian Peninsula, mostly above 1000 m elevation, and form the dominant fodder vegetation. At lower and middle altitudes, fodder is usually tree and shrub browse, whilst at higher altitudes, grasses and herbs are the main fodder vegetation [23].

Wadis, found in arid and semi-arid regions of the Middle East and N Africa, are dry river beds or valleys that are dry but wet during the rainy season. Some wadis that have pools of permanent water fed by springs from surrounding mountains are referred to as oases. The wadis in the Arabian Peninsula are the most inhabited areas. These are extensively cultivated with date gardens and subsistence agriculture of fruit and vegetables. The native vegetation of the wadis is determined by the nature of the drainage system, the groundwater level, the sediment type, and overflow frequency. They are, in general, dominated by *Tamarix* spp. And, depending on the region of the Arabian Peninsula, are inhabited by a variety of different species: a Mediterranean–Sub Saharan type with *Retama raetam*, *Nerium oleander,* and *Pistacia atlantica*, a North Arabian *Vachellia* pseudo-savanna with Saharan shrubs in the understorey, a southeastern community type with *Saccharum griffithii*, *Nerium oleander,* and *Plumbago arabica,* or a southwestern riparian forest of tropical character which is rich in Sudanian vegetation types. Throughout the Peninsula, the wadis have been greatly reformed due to development, cultivation, and grazing by livestock. The native vegetation there has mostly disappeared, and plant species that are unpalatable to livestock such as *Rhazya stricta*, *Peganum harmala*, and *Zilla spinosa* form the dominant plants [23].

**Figure 4:**
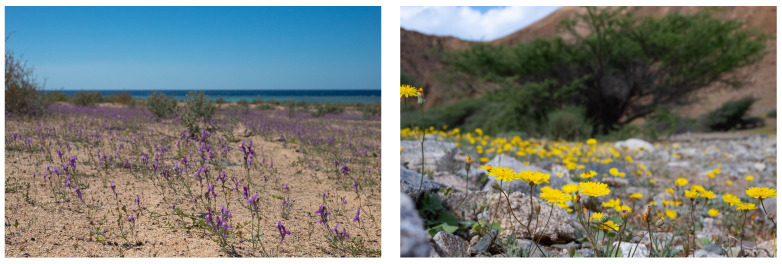
Super blooms of *Linaria haelava* and *Picris babylonica*. Photo © E. Hopkins.

Characteristic to the Arabian Peninsula are its sand dune deserts that occupy almost a third of the land area of the Peninsula. The sands are largely unstable and poor in nutrients, but compared to silty or clay soils, they have a high porosity and permeability with deep moisture penetration. Evaporation of the upper few centimeters of sand is rapid but much slower below, allowing plant growth of a few species, such as *Calligonum comosum,* whose extensive horizontal root system enables it to make optimum use of moisture. Tussock grasses, such as *Centropodia*, and sedges, such as *Cyperus aucheri,* are able to grow on the soft sands of slip faces. Rhizosheaths characteristic of the root systems of desert perennial grasses such as *Stipagrostis plumosa* also play an important role in water absorption and nitrogen fixing in sandy soils. Nitrogen fixing bacteria have been found associated with the root sheaths formed of matted root hairs and sand grains held by secreted mucilage [24,25]. Annuals, such as *Aizoon canariensis*, *Asphodelus fistulosus*, *Boerhavia diffusa*, *Cleome* spp., *Diplotaxis harra*, *Gypsophila capillaris*, *Hippocrepis constricta*, *Plantago* spp., *Reichardia tingitana*, *Reseda muricata*, *Rumex vesicarius*, *Polycarpaea robbairea,* and *Zygophyllum simplex* are common after winter or spring rain, exhibiting a typical desert ephemeral life cycle with rapid germination, development, and flowering and fruiting [26]. Mass flowering or super blooms are uncommon in the Arabian Peninsula, but after a heavy rainfall event in NW Saudi Arabia in March 2024, mass flowering of a few plant species were observed on the coastal hills. The species included the annuals *Linaria haelava*, *Picris asplenioides*, *P. babylonica*, *Polycarpaea robbairea,* and *Zygophyllum simplex* [pers. Comm. 2024] (Figure 4). In February 2023, heavy winter rain caused a super bloom of *Horwoodia dicksoniae* in the Rafha area near the Saudi Arabian border with Iraq [27].

The diverse geomorphology of the coastline greatly influences the distribution of species on the coasts and coastal sabkhas of the Arabian Peninsula. Coastal and sabkha vegetation are poor in species diversity and show marked zonation patterns. Inland sabkhas are virtually unvegetated, with a few fringing halophytic species. *Avicennia marina*, the native mangrove species, has a patchy distribution all along the coasts of the Peninsula [10].

## 3. Biogeography

The Arabian Peninsula is unique due to its geographical position and diverse biogeographic realms, which include the Palearctic, Afrotropical, and Indomalayan regions. Many of the relictual plants of Mediterranean, Eurasian, or African origin survive in small populations in restricted localities on the summits and gorges of the mountains of southern and western Arabia, and on the northern mountains of Oman. Such populations play an important role in the conservation and dissemination of genetic material, as well as in the evolution of new forms. The refugia habitats support plants that have remained in the area from various penetrations of flora in the remote past and may hold the genetic resilience to survive present climatic changes.

Despite the presence of the wild olive on the highest mountains of Yemen, Saudi Arabia, and Oman, and *Pistacia khinjuk* on the mountains of NW Saudi Arabia, the Arabian Peninsula is not a center of diversity for crop wild relatives of agricultural use. However, some relictual species and species with disjunct distribution patterns provide genetic material as a resilient genetic resource for tolerance of drought and salinity that can be used for crop improvement. An example of a drought-resistant species such as *Abelmoschus esculentus,* found wild in southern Oman, probably represents a wild strain and progenitor of the modern cultivated varieties [28].

Distribution patterns of the present flora of the Arabian Peninsula reflect its past floristic ties with African, Iranian, and SW Mediterranean floristic elements. The SW region of Arabia, the escarpment mountains of SW Saudi Arabia, Yemen, and Dhofar, forms an integral part of Africa, both in geological and biogeographical terms. The majority of the species found below 1500 m elevation in southern Arabia also occur in NE and E Africa [1,2]. These include genera with several species such as *Vachellia* (*V. asak*, *V. etbaica*, *V. hamulosa*, and *V. oerfota*), *Commiphora* (*C. foliacea*, *C. gileadensis*, *C. kua*, *C. myrrha*, and *C. schimperi*), *Gymnosporia* (*G. arbutifolia*, *G. obscura*, *G. senegalensis*, and *G. undata*), *Lannea* (*L. malifolia* and *L. triphylla*), and *Turraea* (*T. holstii* and *T. parviflora*), as well as *Boswellia sacra*, *Cadaba baccarinii*, *Premna resinosa*, and *Rhigozum somalense*. Several Afromontane genera such as *Anagyris*, *Ceratonia*, *Juniperus*, *Iris*, *Kniphofia*, *Myrsine*, *Pavetta*, and *Teclea* reach the SW region of Saudi Arabia, Yemen, and southern Oman. It has been suggested that these Ethiopian–Somalian taxa migrated from Africa and became established at a time when the climate was relatively more moist than today and the desert areas between the northern and southern regions of Arabia narrower [2].

**Figure 5:**
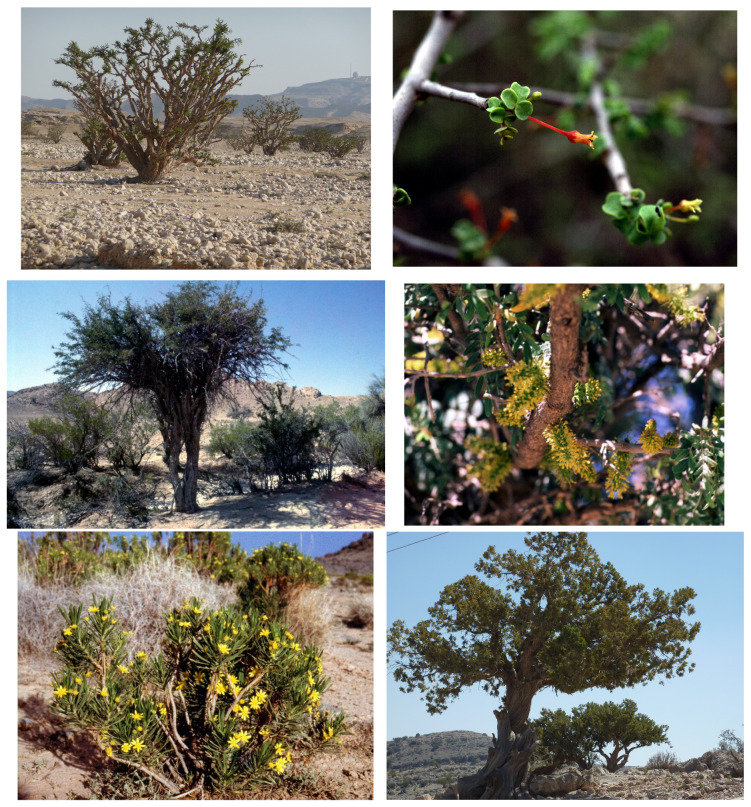
**Top**: **L**–**R**. *Boswellia sacra* (Oman, photo © S. Breckle); *Commiphora foliacea* (Oman). **Middle**: *Ceratonia oreothauma* subsp. *oreothauma*, habit and male flowers (Oman). **Below**: **L**–**R**. *Euryops arabicus* (Oman); *Juniperus seravaschanica* (Oman, photo © S. Breckle). Photos: © S.A. Ghazanfar (other than those of S. Breckle).

Floral similarities between the montane regions of northern Oman, UAE, and Baluchistan and SW Iran suggest the existence of plant communities with *Juniperus seravaschanica*, *Helianthemum lippii*, *Ephedra pachyclada*, and associated species such as *Cymbopogon schoenanthus*, *Lonicera hypoleuca*, *Sageretia thea,* and *Berberis baluchistanica. Prunus arabica* migrated from Baluchistan and SW Iran across the Persian Gulf during the humid period some 30,000 to 20,000 years BP [29]. Over the last arid phase (between 11,000 and 4000 years BP), distribution declined and became restricted to favorable areas at high elevations in the northern mountains of Oman. *Ceratonia oreothauma* subsp. *Oreothauma*, endemic to the summit areas of the eastern Hajar mountains, is a Nubo-Sindian relic confined to a few localities such as gorges and depressions that are relatively more moist [30] (Figure 5).

Recent surveys on mountain summits of NW Saudi Arabia show these to be a bioclimatic refuge for relict Mediterranean and Irano-Turanian species. The cool and relatively humid conditions support plant communities with open evergreen woodlands of scattered *Juniperus phoenicea*, with *Pistacia khinjuk*, *Retama raetam*, *Moringa peregrina*, *Olea europaea* subsp. *cuspidata*, *Dodonaea angustifolia*, and *Globularia arabica*, and a dense ground layer of Irano-Turanian taxa such as *Artemisia sieberi* and *Astracantha echinus* subsp. *arabica*. Relictual Mediterranean and Irano-Turanian taxa include *Thymus decussatus*, *Phlomis brachyodon*, *Atraphaxis spinosa*, *Verbascum decaisneanum*, *Dianthus sinaicus*, *Daphne linearifolia*, *Ephedra pachyclada* var. *sinaica*, *Ajuga chamaepitys* subsp. *tridactylites*, *Colutea istria*, *Echinops glaberrimus*, and *Scorzonera intricata. Pistacia khinjuk*, an Irano-Turanian species, probably migrated before the Pleistocene from the Kurdo-Zagrosian mountains (together with other species such as *Prunus korshinskyi*, *Pistacia atlantica*, *Astragalus echinus,* and *Retama raetam*). *Pistacia khinjuk* is rare in Saudi Arabia and is found at high elevations (above 1500 m) in the mountains of NW Saudi Arabia; it has its southernmost distribution in the Sinai (Egypt) and Sudan [31].

Borrell et al. [32] studied the distribution of endemic plant species in central Oman, which is classified as one of the centers of endemism in Oman [33,34]. They showed that the central desert of Oman presented a southern Arabian Pleistocene refugium, with a high number of endemic species within a narrow monsoon-influenced region. They suggest the vegetation there is a relict of an earlier, more mesic period.

The Amaranthaceae genera present in the Arabian Peninsula, such as *Anabasis setifera*, *Anabasis articulata*, *Atriplex leucoclada*, *Noaea mucronata,* and *Caroxylon villosum,* are examples of the Sahara–Arabian desert flora from the Mesogean stock (of the region that developed into the Mediterranean Sea) [2]. A single Afromontane species, *Euryops jaberiana,* is endemic to the NW mountains of Saudi Arabia [35]. A second species, *Euryops arabicus,* is found in the Arabian Peninsula in the mountains of northern Oman and Yemen and in Djibuti and Somalia in NE Africa [36,37](Figure 5). *Euryops jaberiana*, *E. arabicus,* and *E. socotranus* (the latter two found also in Socotra and Ethiopia) are the only three taxa found outside Africa.

## 4. Conservation

The widespread and accelerated loss of biological diversity and the magnitude of this loss to the region’s economy and culture is well recognized. The Arabian Peninsula has been relatively slow to recognize and act upon its loss of biodiversity, but over the last two decades, several measures have been taken to address this problem. Most of the countries of the Arabian Peninsula are signatory to the Convention of Biological Diversity (CBD) and have taken active participation at the Convention of Parties (COP) meetings [38]. A target of establishing 17% of protected areas in a country is being considered seriously and the conservation of plant genetic resources, primarily through the establishment of seed banks, is being addressed.

**Figure 6:**
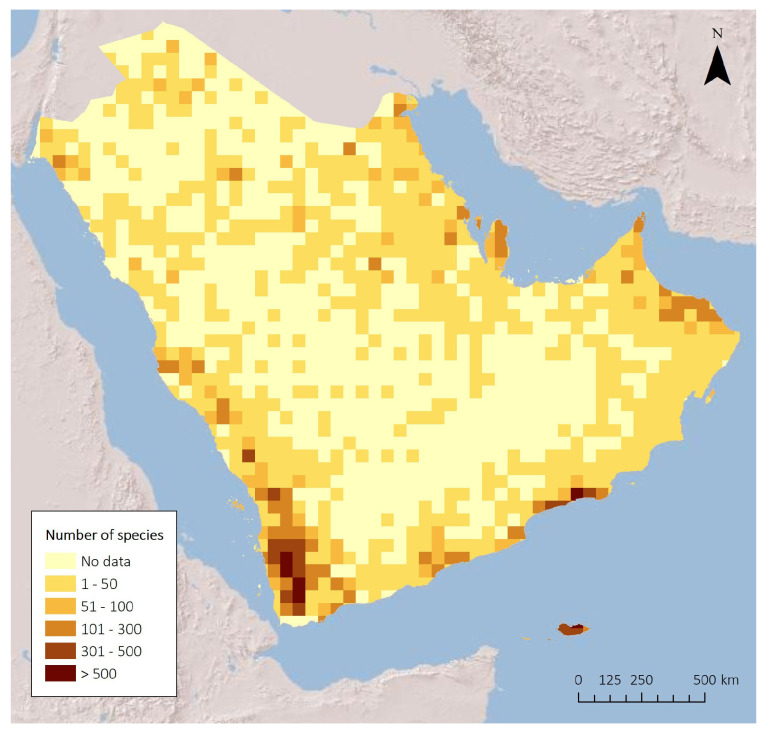
Species richness in the Arabian Peninsula where most of the endemic species are found. From Forrest and Neale, The Conservation Status of the Plants of the Arabian Peninsula: Endemic Taxa, Trees, and Aloes. Environment and Protected Areas Authority, Sharjah, UAE, 2023 [32].

A recently available Red List for the plants of the Arabian Peninsula summarizes the conservation status of some plants [39]. The list shows that of a total of 797 taxa that were formally assessed (according to the IUCN guidelines), 232 (29%) were found to be threatened. Several biodiversity hotspots are recognized and it can be clearly seen that the number of species, and especially the number of endemic species, are concentrated in specific localities (Figure 6). The biodiverse areas are (1) the southwest mountains of Yemen, which are part of the Eastern Afromontane Biodiversity Hotspot, (2) several Regional Centers of Plant Diversity, viz., the fog oasis of Dhofar, southern Oman, the Harrat Al-Harrah Highlands of southwestern Arabia, Hadramaut, Jebel Areys, and the island of Socotra. The Socotra Archipelago is also a UNESCO World Heritage Site, and a Man and Biosphere Reserve recognizing globally for its unique natural heritage. There are also several Ramsar sites across the Peninsula, e.g., Hawar Islands of Bahrain and the Mubarak Al-Kabeer Reserve in Kuwait.

Official or unpublished Regional Red Lists of plants are now available for Oman [40], the UAE [41], and Saudi Arabia [42], but a Red List assessing all plants of the Arabian Peninsula is not yet available. Several countries of the Arabian Peninsula have officially designated nature reserves to conserve and protect the country’s floral and faunal diversity and to carry out plant surveys to document and map their floral diversity.

There is also now a concentrated effort to document the plant genetic resources (PGRs) of the Arabian Peninsula. PGRs, especially crop wild relatives (CWRs), provide a major resource of food for present and future generations and provide promising alternatives in developing new and desirable crop varieties through different plant breeding approaches. Countries of the Arabian Peninsula, such as Saudi Arabia, have been a part of the Treaty on PGRs for Food and Agriculture, which aims to conserve, utilize, and share the benefits of available genetic diversity and resources [43]. An analysis of the flora from the Arabian Peninsula shows that there are over 400 wild relatives of some 70 food and forage crops. These species, because of their survival and adaptation to the extreme arid climate of the Arabian Peninsula, are an important source of genetic resource which can be used for crop improvement programs directed towards climate change [44,45]. More recently, seeds of five crops of high value as food were collected in the southeastern province of Jazan in Saudi Arabia and stored in seed banks to preserve the genetic diversity of these crops so as to ensure continued cultivation (in situ conservation) [46]. In Oman, the discovery of wild date palms has also been used to trace the domestication history of the date palm [47,48].

## 5. Summary

The Arabian Peninsula is unique due to its geographical position and diverse biogeographic realms, which include the Palearctic, Afrotropical, and Indomalayan regions. It is home to a rich and diverse flora and has several species that are remnants of a once widespread distribution. The mountains create cooler and more moderate climates, leading to the formation of special ecosystems that serve as refugia for plant and animal species. There is a high level of plant species that are unique to the region. Conservation programs to preserve and conserve the plant diversity of the countries of the Arabian Peninsula have been slow, but more recently, conservation efforts have been accelerated. Efforts have concentrated on producing plant Red Lists, demarking important plant areas, and designating nature reserves. Due to its long history of human habitation and subsistence agriculture, particularly in the mountainous areas, the Arabian Peninsula possesses unique crop varieties adapted to extreme arid climates, making them important genetic resources for the future in the face of climate change.

## Figures and Tables

**Table 1:** Species richness and endemism in the countries of the Arabian Peninsula (adapted from Miller & Cope, 1996 [17]).

Country	Area (km^2^)	Taxa (~)	Endemic Taxa
Yemen	555,000	2580	458 (17.7%)
Saudi Arabia	2.25 million	2250	246 (10.9%)
Oman	309.5000	1440	69 (4.79)
United Arab Emirates	83.6000	598	0
Kuwait	17.82000	404	0
Qatar	11.44000	371	0
Bahrain	786.5	323	0

## Data Availability

The original contributions presented in the study are included in the References at the end of this article. Further inquiries can be directed to the corresponding author/s. No new data were created or analyzed in this study. Data sharing is not applicable to this article.
